# Impact of vaccination and variants of concern on long COVID clinical phenotypes

**DOI:** 10.1186/s12879-023-08783-y

**Published:** 2023-11-16

**Authors:** Grace Kenny, Kathleen McCann, Conor O’Brien, Cathal O’Broin, Willard Tinago, Obada Yousif, Tessa O’Gorman, Aoife G. Cotter, John S. Lambert, Eoin R. Feeney, Eoghan de Barra, Corinna Sadlier, Alan Landay, Peter Doran, Stefano Savinelli, Patrick W. G. Mallon, The All Ireland Infectious Diseases Cohort Study, Rachel MacCann, Alejandro Garcia Leon, Sarah Miles, Dana Alalwan, Riya Negi, Eavan Muldoon, Gerard Sheehan, Tara McGinty, Sandra Green, Kelly Leamy, Christine Kelly, Eoin de Barra, Samuel McConkey, Killain Hurley, Imran Sulaiman, Mary Horgan, Joseph Eustace, Tommy Bracken, Bryan Whelan, Justin Low, Bairbre McNicholas, Garry Courtney, Patrick Gavin

**Affiliations:** 1 Centre for Experimental Pathogen Host Research, University College Dublin, Belfield Dublin 4, Ireland; 2 St Vincent’s University Hospital, Dublin, Ireland; 3 School of Medicine, University College Dublin, Dublin, Ireland; 4 Wexford General Hospital, Wexford, Ireland; 5 Mater Misericordiae University Hospital, Dublin, Ireland; 6 Beaumont Hospital, Beaumont, Dublin 9, Ireland; 7 Department of International Health and Tropical Medicine, Royal College of Surgeons in Ireland, Dublin, Ireland; 8 Department of Infectious Diseases, Cork University Hospital, Wilton, Cork Ireland; 9 Department of Internal Medicine, Rush University, Chicago, IL USA; 10 Clinical Trials Institute, University of Galway, Galway, Ireland

**Keywords:** SARS-CoV-2 variants, Long COVID, Post-Acute Sequelae of SARS-CoV-2 infection

## Abstract

**Background:**

Defining patterns of symptoms in long COVID is necessary to advance therapies for this heterogeneous condition. Here we aimed to describe clusters of symptoms in individuals with long COVID and explore the impact of the emergence of variants of concern (VOCs) and vaccination on these clusters.

**Methods:**

In a prospective, multi centre cohort study, individuals with symptoms persisting > 4 weeks from acute COVID-19 were divided into two groups based on timing of acute infection; pre-Alpha VOC, denoted wild type (WT) group and post-Alpha VOC (incorporating alpha and delta dominant periods) denoted VOC group. We used multiple correspondence analysis (MCA) and hierarchical clustering in the WT and VOC groups to identify symptom clusters. We then used logistic regression to explore factors associated with individual symptoms.

**Results:**

A total of 417 individuals were included in the analysis, 268 in WT and 149 in VOC groups respectively. In both groups MCA identified three similar clusters; a musculoskeletal (MSK) cluster characterised by joint pain and myalgia, a cardiorespiratory cluster and a less symptomatic cluster. Differences in characteristic symptoms were only seen in the cardiorespiratory cluster where a decrease in the frequency of palpitations (10% vs 34% *p* = 0.008) and an increase in cough (63% vs 17% *p* < 0.001) in the VOC compared to WT groups was observed. Analysis of the frequency of individual symptoms showed significantly lower frequency of both chest pain (25% vs 39% *p* = 0.004) and palpitations (12% vs 32% *p* < 0.001) in the VOC group compared to the WT group. In adjusted analysis being in the VOC group was significantly associated with a lower odds of both chest pain and palpitations, but vaccination was not associated with these symptoms.

**Conclusion:**

This study suggests changes in long COVID phenotype in individuals infected later in the pandemic, with less palpitations and chest pain reported. Adjusted analyses suggest that these effects are mediated through introduction of variants rather than an effect from vaccination.

**Supplementary Information:**

The online version contains supplementary material available at 10.1186/s12879-023-08783-y.

## Introduction

The management of long COVID remains one of the most challenging aspects of the COVID-19 pandemic [1]. Long COVID is used to describe a number of symptoms that persist beyond the acute viral illness, but criteria to differentiate these into distinct clinical categories are lacking. While it is increasingly acknowledged long COVID is not one distinct entity, frequently studies exploring the pathogenesis or long COVID attempt to overcome this heterogeneity by correlating individual symptoms with specific immune abnormalities [2], running the risk of multiple comparisons and potentially erroneous conclusions. Identification of consistent patterns of symptoms in long COVID that could be used to guide therapeutic and translational studies is urgently needed. We and others have used cluster analysis to identify clinical phenotypes of long COVID [3–5], but a variety of factors have emerged that may impact these patterns. Specifically, vaccination against SARS-CoV-2 has been associated with both a lower risk of developing long COVID [6] and an improvement in long COVID symptoms in observational studies [7]. In addition, variants of concern (VOCs) have emerged which differ in virulence [8] and transmission dynamics [9] to wild-type SARS-CoV-2 but the impact of VOCs on post-acute symptoms has not been determined. Here, we aimed to identify phenotypes of long COVID in individuals infected prior to and subsequent to the emergence of VOCs, explore changes in long COVID phenotype across these time periods, and the association of factors including VOCs and vaccination with these changes.

## Methods

The All Ireland Infectious Diseases (AIID) cohort is a prospective, multicentre, observational cohort study recruiting individuals presenting with issues pertaining to infectious diseases in participating hospitals in Ireland, described in detail elsewhere [10]. The AIID cohort study was approved in line with national and European regulations on health research by the St Vincent’s Hospital group Research Ethics committee and the National Research Ethics Committee for COVID-19 in Ireland. All study procedures adhered to required guidelines. Participants provide written informed consent for collection of data on demographics, clinical characteristics and investigations undertaken as part of routine care. For this study, individuals were included if they were attending for assessment of long COVID, had PCR-confirmed COVID-19, and were still symptomatic at least 4 weeks post onset of acute symptoms.

The details of the long COVID assessments have been described in detail elsewhere [3]. Briefly, 19 symptoms, maximum acute disease severity as graded by the World Health Organisation (WHO) scale [11], Medical Research Council (MRC) dyspnea scale [12] and Short Form-36 (SF-36) [13] score were assessed at each clinic visit using a standardised proforma. Participants self-completed SF-36 questionnaires, while other components were assessed by structured interview and review of medical records. The MRC dyspnea scale is a validated 5 point scale that assesses functional disability due to dyspnea, ranging from no disability (point 1) to dyspnea limiting basic activities of daily living (point 5). The SF-36 survey is a generic measure of health status and quality of life, evaluating an individual’s perception of their performance in 8 domains, and has shown to be a reliable, valid and sensitive measure of health status in a variety of settings. Additional investigations were performed if determined to be indicated by the assessing clinician. For analysis we included only symptoms present in at least 10% of individuals, to maintain an appropriate number of variables for the sample size [14].

As sequencing data was not available, individuals were allocated to two groups based on timing of acute COVID-19. The first period (“wild-type” (WT) period) included those with onset of symptoms prior to 26^th^ December 2020 when the alpha variant became dominant in Ireland, and the VOC period individuals with symptoms after this date. The delta variant became dominant in April 2021 and the Omicron variant in December 2021 in Ireland [15]. For individuals who reported more than one infection prior to their review, we allocated group based on the infection that the participant attributed their long COVID symptoms to.

### Statistical analysis

Categorical variables were summarised using number and percentage and continuous variables with median and interquartile range (IQR). Cluster analysis was performed as described in detail previously [3]. Briefly, we used multiple correspondence analysis (MCA) to remove dimensionality from the dataset. MCA is a principal component analysis method that transforms categorical data into coordinates in multidimensional space (using χ2 distance between coordinates so similar individuals lie closer together). The smallest number of dimensions that account for the largest total explained variance are retained for further analysis, resulting in a reduction in the number of variables needed to summarize the data. Then, agglomerative hierarchical clustering was performed on the results of the MCA, using squared Euclidean distance and Ward’s minimum variance linkage. The number of clusters was selected at the partition where there was the greatest within cluster loss of inertia. We used heatmaps to visualise the prevalence of individual symptoms within each cluster and compared continuous variables and categorical variables with Kruskal–Wallis and chi square test respectively. As this was an exploratory analysis, we did not correct for multiple comparisons [16].

Univariate and multivariable logistic regression models were constructed to explore the association of infection period and vaccination status on individual symptoms. In multivariable models, we adjusted for age, sex, ethnicity, WHO acute disease severity and time from symptom onset. All analysis was carried out using R version 4.2.1.

## Results

### Participant characteristics

From 1^st^ March 2020 to 10^th^ March 2022, 2,392 individuals were recruited to the AIID cohort study, of these 1,644 were recruited for conditions other than post COVID review. Of the 748 individuals recruited at post COVID review, 519 were still experiencing symptoms. Of these, 52 did not have COVID-19 confirmed by PCR and 23 were seen < 4 weeks from symptom onset. Of the remaining 444, 417 had complete data and were included in this analysis. Of these, 268 were infected prior to the introduction of the alpha variant in Ireland (wild-type (WT) period) and 149 after the alpha variant became dominant (VOC period). Within the VOC period, 124 were infected in alpha dominant and 25 in delta dominant periods. Two individuals infected within the WT period had a second infection in the VOC period prior to review, and one individual infected in the VOC period reported a prior infection in the WT period that did not lead to long COVID symptoms.

Participant demographics are shown in Table 1. Basic demographics were similar across both periods, median (IQR) age was 45 (35–55) years in the WT period and 47 (34–57) years in the VOC period (*p* = 0.2). 73% of individuals were female in both periods (*n* = 196 and *n* = 109 in WT and VOC periods respectively, *p* = 0.9), and the majority experienced a mild acute illness (83% in WT and 75% in VOC, *p* = 0.07). There were no significant differences in ethnicity, BMI or comorbidities across time periods, but significantly more individuals were vaccinated both prior to infection and after acute infection but prior to review in the VOC compared to the WT period (0 (0%) and 15 (11%) vaccinated at time of infection (*p* < 0.001) and 60 (25%) and 97 (75%) vaccinated by the time of clinic review (*p* < 0.001) in the WT and VOC periods respectively.

**Table 1:** Participant demographics

	**Whole cohort (*n* = 417)**	**WT (*n* = 268)**	**VOC (*n* = 149)**	***P* value**
**Age (years)**	45 (35–55)	43 (36–54)	47 (34–57)	0.2
**Sex (F)**	307 (73)	196 (73)	109 (73)	0.9
**Ethnicity (Caucasian)**	352 (84)	222 (84)	129 (87)	0.31
**BMI (kg/m**^**2**^**)**	28 (24–32)	28 (24 – 32)	28 (24 – 33)	0.6
**HCW**	215 (51)	163 (61)	51 (35)	< 0.001
**Smoking**	19 (4)	12 (4.5)	7 (4.7)	0.9
**Alcohol**	125 (30)	80 (30)	45 (30)	0.9
**Comorbidity (Any)**	243 (56)	152 (59)	89 (60)	0.9
**Hypertension**	74 (18)	45 (18)	28 (19)	0.8
**Diabetes**	23 (5)	14 (5.5)	9 (6.1)	0.9
**Respiratory**	78 (19)	45 (18)	32 (22)	0.4
**-Asthma**	62 (15)	36 (13)	25 (17)	0.43
**Cardiac**	21 (5)	13 (5.1)	8 (5.4)	0.9
**Immunosuppression**	5 (1)	2 (0.8)	3 (2)	0.5
**Psychiatric**	10 (2)	8 (3)	2 (1)	0.47
**Vaccinated at time of infection**	15 (3)	0 (0)	15 (11)	< 0.001
**Vaccinated at time of review**	157 (37)	60 (25)	97 (75)	< 0.001
**Time from symptom onset (weeks)**	22 (13–35)	24 (16–38)	18 (10–31)	< 0.001
**Mild initial disease severity**	330 (79)	217 (83)	112 (75)	0.07
**Hospitalised at acute infection**	129 (31)	75 (28)	54 (36)	0.11

*Legend*: *BMI* Body mass index. *HCW* Healthcare worker. Continuous variables compared with Kruskal–Wallis test and categorical variables by chi square test. Continuous data are median (IQR) and categorical data number (%)

### Symptom clusters

**Fig. 1:**
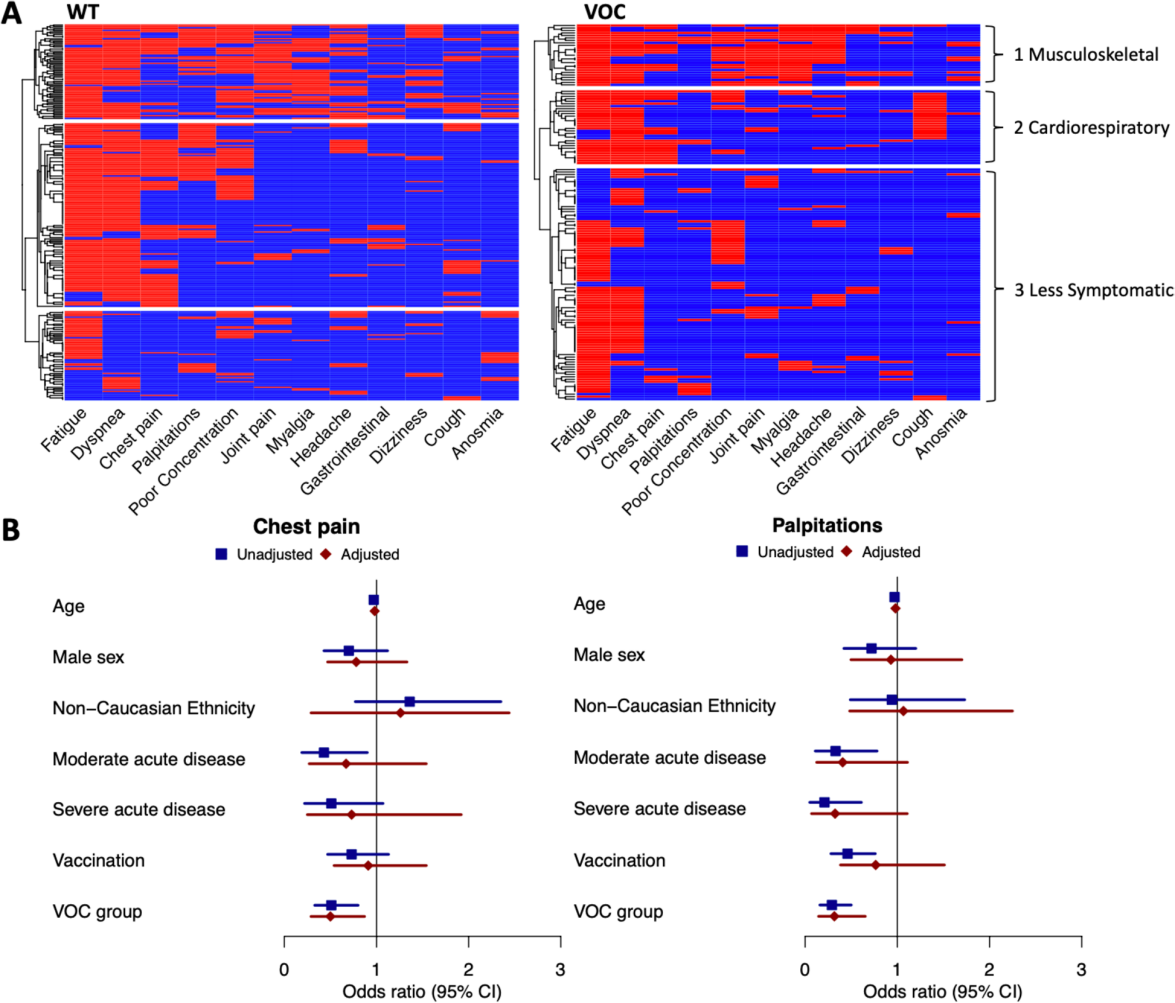
Heatmaps of self-reported symptom clusters and forest plots showing odds of chest pain and palpitations associated with individual symptoms. Legend: **A** Heatmaps demonstrating hierarchical clustering on self-reported symptoms. WT group (1^st^ March 2020-25^th^ December 2020) is shown on the left and VOC group (26^th^ December 2020- 1^st^ March 2022) on the right. VOC group included 124 individuals with acute infection in the alpha variant dominant period and 25 infected in the delta dominant period. In both groups the top cluster is a musculoskeletal cluster (*n* = 69 (26%) in WT and *n* = 23 (15%) in VOC), the middle is a cardiorespiratory cluster (*n* = 134 (50%) in WT and *n* = 30 (20%) in VOC) and the bottom is a less symptomatic cluster (*n* = 65 (24%) in WT and *n* = 94 (63%) in VOC). **B** Forest plot showing the unadjusted and adjusted odds ratios for reporting chest pain and palpitations

In both WT and VOC periods, multiple correspondence analysis and hierarchical clustering revealed three distinct symptom clusters (Fig. 1A). Table S1 shows the cluster symptom profiles across groups.

The first cluster (*n* = 69 (26%) in WT and *n* = 25 (17%) in VOC) we labelled a musculoskeletal (MSK)/pain cluster. This cluster was characterised by the highest number of symptoms (median (IQR) 6 (5–7) per individual in both time periods), with joint pain and myalgia in particular overrepresented compared to the other two clusters (joint pain present in 74% and 84% and myalgia in 64% and 92% in the WT and VOC period respectively). Headache (present in 54% and 68% in the WT and VOC periods), poor concentration (present in 68% and 76% in the WT and VOC periods) and GI symptoms (present in 19% and 32% in the WT and VOC periods) were also overrepresented in this cluster. This cluster reported the greatest functional impact compared to other clusters across both time periods (Table S2), with the longest time missed from work (median (IQR) 11.5 (4–18.75) weeks in the WT and 12 (4–32) weeks in the VOC period), and worst SF-36 scores in the domains of physical functioning, pain, general health and social functioning.

Individuals in Cluster 2 self-reported a median of 3 (IQR 4–5) symptoms in the WT period and 4 (IQR 3–5) in the VOC period. Characteristic symptoms were cardiorespiratory in both periods. Dyspnea (present in 91% and 97% in the WT and VOC periods) and chest pain (present in 57% and 60% in the WT and VOC periods) were common in this cluster, however the proportion of individuals in this cluster reporting palpitations was significantly higher in the WT period (34% vs 10% *p* = 0.008), and cough significantly lower (17% vs 63% *p* < 0.001) in the WT than the VOC period. Overall this cluster incorporated fewer individuals within the VOC period compared to the WT period (50% of WT and 20% of VOC period, *p* < 0.001), and had less functional impact in terms of illness associated work absence in the VOC compared to the WT period (median (IQR) 8 (3–17) weeks missed in the WT period and 4 (0–7) in the VOC period, *p* = 0.007) (Table S4).

The third cluster reported less overall symptoms (median 2 (IQR 1–3) per patient across both time periods. In the WT period, anosmia was more frequent in this cluster than the other two clusters, (present in 25%), whereas in the VOC period no single symptom predominated. In line with the lower frequency of symptoms, this cluster had the least functional impact across both periods, with the best breathlessness scores as measured by the MRC dyspnea scale, and the highest health related quality of life in the SF36 domains of physical functioning, pain, general health and social functioning in both periods (Table S4).

### Vital signs and investigations

Three hundred thirteen (75%) of individuals had resting vital signs available. Median (IQR) heart rate was 74 (66–85) for the overall cohort and there was no difference between WT and VOC periods (median (IQR) heart rate 74 (65–85) in the WT period, 74 (66–84) in the VOC period, *p* = 0.72). Given the change in subjective palpitations within the cardiorespiratory cluster across WT and VOC periods we next looked specifically at vital signs within this cluster. 102 (76% of the WT and 21 (70%) of the VOC cardiorespiratory cluster had resting vital signs available and 72 (54%) of the WT and 20 (66%) of the VOC cardiorespiratory cluster had orthostatic vital signs available. There was no difference in heart rate in the WT cardiorespiratory cluster at rest (median (IQR) resting HR 75 (67–85) in WT and 69 (65–80) in VOC period *p* = 0.35), or at 1, 3 and 5 min standing (median (IQR) HR at 1 min standing; 85 (78–96) in WT and 78 (72–89) in VOC, at 3 min standing; 86 (78–97) in WT and 79 (72–90) in VOC, at 5 min standing; 88 (79–97) in WT and 79 (75–91) in VOC *p* = 0.19) compared to the VOC cardiorespiratory cluster.

Looking next at cardiac imaging, results of 64 echocardiograms were available, 56 in the WT period (21% of WT period) and 8 in the VOC period (5% of VOC period). 9 individuals had findings consistent with pericarditis or a pericardial effusion, 8 within the WT period and 1 in the VOC period 7 (78%) of these were in individuals in the cardiorespiratory cluster.

Results of 40 cardiac MRIs were available, 34 in the WT (13% of WT period) and 6 in the VOC period (4% of VOC period). There were 4 cardiac MRI confirmed diagnoses of myocarditis, all of these were in individuals within the cardiorespiratory cluster in the WT period.

### Change in symptoms across periods of infection

Given the change in characteristic symptoms in the cardiorespiratory cluster, we next compared the frequency of all symptoms across the full cohort during WT and VOC periods to determine if an overall change in symptom frequency was mediating this difference. There were significant differences only in the frequency of palpitations (32% vs 12% in the WT vs VOC groups *p* < 0.0001) and chest pain (39% vs 25% *p* = 0.004) but not cough (16% vs 15% *p* = 0.9), or any other symptom.

Looking first at chest pain, in univariate analysis, along with being in the VOC group (OR 0.51 (95% CI 0.33–0.8), *p* = 0.003), increasing age (OR 0.97 (95% CI 0.96–0.99), *p* < 0.001), and moderate severity acute COVID-19 (OR 0.43 (95% CI 0.19–0.9), *p* = 0.033) were significantly associated with a reduced odds of chest pain (Fig. 1B, Table S3). There was no association between vaccination, either at the time of initial infection or at the time of review and odds of reporting chest pain. In adjusted analysis only older age (OR 0.98 (95% CI 0.96–0.99), *p* = 0.02) and being in the VOC group (OR 0.5 (95% CI 0.29–0.87), *p* = 0.02) remained significantly associated with a reduced odds of reporting chest pain.

Similarly, regarding palpitations, in univariate analysis being in the VOC group (OR 0.29 (95% CI 0.16–0.5), *p* < 0.0001), older age (OR 0.97 (95% CI 0.96–0.99), *p* = 0.02), and more severe acute disease (moderate disease OR 0.33 (95% CI 0.11–0.78), *p* = 0.022, severe disease OR 0.21 (95% CI 0.05–0.61), *p* = 0.011) were associated with a reduced odds of reporting palpitations. Interestingly, vaccination at the time of review (OR 0.46 (95% CI 0.28–0.76), *p* = 0.003) (Fig. 1B, Table S4), but not at the time of infection (OR 0.21 (95% CI 0.01–1.04), *p* = 0.13) was also associated with a reduced odds of reporting palpitations. However in fully adjusted analysis, only being in the VOC group (OR 0.37 (95% CI 0.18–0.71), *p* = 0.004), but not vaccination (OR 0.59 (95% CI 0.33–1.07), *p* = 0.08), remained significantly associated with a reduced odds of palpitations.

## Discussion

In this analysis we demonstrate three similar symptom clusters in two, independent groups of participants with confirmed SARS-CoV-2 at different periods of the pandemic. Validation of these clusters across independent time points suggests distinct clinical phenotypes that may share underlying mechanisms, while variation in reported symptoms suggests modification of long COVID over time.

Similarly, we observed a decrease in cardiac symptoms of chest pain and palpitations between the pre and post VOC periods. This association between infection period and lower odds of palpitations or chest pain remained robust in fully adjusted models, suggesting this change may be mediated by change in the infecting variant.

Interestingly, while the symptoms contributing to the clusters were broadly similar across the two time periods, we observed a shift from cardiac to respiratory predominant symptoms within the cardiorespiratory cluster between WT and VOC periods, with a decrease in palpitations, an increase in cough but persistent dyspnea. There have been a number of studies focused on phenotyping long COVID. While these differ in data source, analytic approach and symptoms included, an MSK/pain dominant, cardiorespiratory and less symptomatic cluster have been found in other studies [5, 17–20]. However, few studies have examined the change in long COVID phenotype with infection period. One study [4] using smartphone application data examined long COVID symptom profiles across WT, alpha and delta waves. While this study demonstrated two clusters similar to the cardiorespiratory cluster and musculoskeletal cluster observed here across all periods, it found different numbers of clusters across variants due to the inclusion of less frequently reported symptoms, limiting the ability to examine changes in the consistent phenotypes. This study also observed a decrease in size of the cardiorespiratory cluster with the introduction of VOCs. The authors postulate that this reflects a reduction in lung damage with the introduction of vaccines, however physician assessed acute disease severity available in this study allowed us to test this hypothesis, finding timing of infection, rather than disease severity or vaccination may mediate this reduction in cardiac symptoms.

We report a decrease in cardiac symptoms with the introduction of VOCs that has not been demonstrated elsewhere. Magnusson et al. looked at the difference in long COVID symptoms between only delta and omicron infections, and found no difference in any post COVID outcome between these two variants [21], suggesting the change in cardiac symptoms may be due to the initial adaptations of the alpha variant that have been retained in subsequent variants. For example the spike mutation D614G found in both alpha and delta variants, increased infectivity in the upper but not lower respiratory tract [22], and may have altered viral tropism to cardiac tissues. Atypical cardiac inflammation has been described in individuals with these long COVID symptoms [23]. While we observed fewer diagnoses of pericarditis and myocarditis within the VOC compared to the WT period within this analysis, relatively few individuals had cardiac imaging results available, and the relationship between VOC infection and cardiac MRI findings requires further study.

There has been considerable interest in the effect of vaccination on long COVID symptoms. While most studies have focused on the effect of vaccination prior to infection, a number of observational studies have suggested an improvement in symptoms with vaccination [7, 24]. While we observed an association between vaccination and a reduced odds of reporting palpitations in univariate analysis, this was significantly attenuated after adjustment, while the association between reduced reporting of palpitations and infection in the VOC period persisted. This would suggest that the change in long COVID symptoms was mediated more by time period of infection rather than by vaccination status. This is consistent with observations elsewhere that while vaccination may reduce long COVID incidence [25], in those who develop post-acute symptoms patterns are similar [21], and demonstrates the importance of considering the introduction of VOCs when determining the effect of vaccination on long COVID.

This analysis has limitations. Although there was a distinct time period in Ireland when the alpha variant became dominant, most subjects attended for clinic visits long after the initial infection, therefore sequencing data to confirm the original infecting variant was not available. In addition, an insufficient number of individuals reporting SARS-CoV-2 infection during the Delta dominant period, meant we were unable to analyse these VOC independently, and this analysis included no individuals infected during Omicron dominant periods preventing a more detailed examination of the different common VOC. We included only the twelve most commonly reported symptoms, and may therefore have missed the impact of VOC on less frequently reported symptoms, however we believe including only the most common symptoms improved cluster interpretability compared to some larger studies derived from electronic health records [26]. We do not explore classification methods in this analysis, and further research is needed to identify how to best assign individuals to the most appropriate cluster. Although we report data from a multicentre study, participating hospitals are all within Ireland and further research is needed to replicate these findings in independent cohorts. Cardiac imaging and vital sign data was incomplete, limiting power to detect differences across symptom clusters and variants, and precluding conclusions of the effect of VOCs on these findings.

In summary, this analysis demonstrates three clinical phenotypes of long COVID, observed across two distinct time periods. We observed a change in pattern of symptoms between the two time periods, which were particularly associated with the cardiorespiratory phenotype, driven by infection during the VOC period rather than vaccination. Further research is needed to determine the pathophysiologic basis of these differences.

### Supplementary Information

**Additional file 1: Supplemental Table 1.** Proportion of individuals experiencing symptoms in each cluster in wild type and variant of concern groups. **Supplemental Table 2.** Functional impact across clusters. **Supplemental Table 3.** Univariate and multivariate models demonstration association of factors with self-reported chest pain. **Supplemental table 4.** Univariate and multivariate models demonstration association of factors with self-reported palpitations

## Data Availability

Researchers can apply for access to pseudonymised data by submitting a data access request to the All Ireland Infectious Diseases Cohort steering group. Requests can be sent to the corresponding author, and access will be granted depending on a data protection impact assessment and assessment of the research proposal.
