# Supplementary material for: Impact of vaccination and variants of concern on long COVID clinical phenotypes
Source: BMC Infect Dis. 2023 Nov 16;23:804. doi: 10.1186/s12879-023-08783-y (PMC10655269; doi:10.1186/s12879-023-08783-y)
Supplement: Supplementary file 1 — Additional file 1: Supplemental Table 1. Proportion of individuals experiencing symptoms in each cluster in wild type and variant of concern groups. Supplemental Table 2. Functional impact across clusters. Supplemental Table 3. Univariate and multivariate models demonstration association of factors with self-reported chest pain. Supplemental table 4. Univariate and multivariate models demonstration association of factors with self-reported palpitations [file 12879_2023_8783_MOESM1_ESM.docx]

Supplementary information

**Supplemental table 1 – Proportion of individuals experiencing symptoms in each cluster in wild type and variant of concern groups.**

| WT period | | | |
| --- | --- | --- | --- |
|  | **Musculoskeletal**  **(n = 69)** | **Cardiorespiratory**  **(n = 134)** | **Less symptomatic**  **(n = 65)** |
| Fatigue | 65 (94) | 122 (91) | 37 (57) |
| Dyspnea | 55 (80) | 122 (91) | 12 (18) |
| Chest pain | 27 (39) | 76 (57) | 2 (3.1) |
| Palpitations | 30 (43) | 46 (34) | 10 (15) |
| Poor concentration | 47 (68) | 41 (31) | 17 (26) |
| Joint pain | 51 (74) | 9 (6.7) | 9 (14) |
| Myalgia | 44 (64) | 6 (4.5) | 3 (4.6) |
| Headache | 37 (54) | 19 (14) | 14 (22) |
| Gastrointestinal | 13 (19) | 15 (11) | 2 (3.1) |
| Dizziness | 28 (41) | 6 (4.5) | 8 (12) |
| Cough | 16 (23) | 23 (17) | 3 (4.6) |
| Anosmia | 13 (19) | 3 (2.2) | 16 (25) |
| VOC period | | | |
|  | **Musculoskeletal**  **(n = 25)** | **Cardiorespiratory**  **(n = 30)** | **Less symptomatic**  **(n = 94)** |
| Fatigue | 25 (100) | 26 (87) | 69 (73) |
| Dyspnea | 19 (76) | 29 (97) | 48 (51) |
| Chest pain | 13 (52) | 18 (60) | 6 (6.4) |
| Palpitations | 4 (16) | 3 (10) | 11 (12) |
| Poor concentration | 19 (76) | 9 (30) | 24 (26) |
| Joint pain | 21 (84) | 4 (13) | 14 (15) |
| Myalgia | 23 (92) | 3 (10) | 6 (6.4) |
| Headache | 17 (68) | 4 (13) | 13 (14) |
| Gastrointestinal | 8 (32) | 1 (3.3) | 6 (6.4) |
| Dizziness | 6 (24) | 2 (6.7) | 8 (8.5) |
| Cough | 1 (4) | 19 (63) | 2 (2.1) |
| Anosmia | 7 (28) | 1 (3.3) | 5 (5.3) |

Legend: Data are number (%).

**Supplemental Table 2 – Functional impact across clusters**

| WT period | | | | | | |
| --- | --- | --- | --- | --- | --- | --- |
|  | **Musculoskeletal (n = 69)** | | **Cardiorespiratory (n = 134)** | | **Less symptomatic**  **(n = 65)** | **P value** |
| Time off work (weeks) | 11.5 (4-18.75) | | 8 (3-17) | | 3 (1-11) | 0.001 |
| MRC | 2 (2-3) | | 2 (2-3) | | 1 (1-2) | <0.0001 |
| SF36 scores |  | |  | |  |  |
| Physical functioning | 45 (25-60) | | 47.5 (28.75-70) | | 80 (55-100) | <0.001 |
| Physical Role Limitation | 0 (0-25) | | 0 (0-25) | | 0 (0-50) | 0.06 |
| Emotional Functioning | 53 (45-67) | | 51 (40-63) | | 56 (42-72) | 0.46 |
| Emotional role limitation | 33 (0-100) | | 33 (0-100) | | 50 (0-100) | 0.87 |
| Energy | 25 (15-35) | | 30 (20-38.75) | | 30 (20-46.25) | 0.42 |
| Pain | 45 (22-57) | | 49 (35-77) | | 77 (45-100) | <0.001 |
| General Health | 40 (26-55) | | 50 (35-65) | | 55 (44-75) | 0.002 |
| Social functioning | 37 (25-59) | | 50 (25-62.5) | | 62.5 (47-75) | 0.003 |
| VOC period | | | | | | |
|  | **Musculoskeletal (n = 25)** | **Cardiorespiratory (n = 30)** | | **Less symptomatic**  **(n = 94)** | | **P value** |
| Time off work (weeks) | 12 (4-32) | 4 (0-7) | | 4 (2-12) | | 0.002 |
| MRC | 2 (2-2) | 2 (2-3) | | 1 (1-2) | | 0.006 |
| SF36 scores |  |  | |  | |  |
| Physical functioning | 30 (22-47) | 50 (27-61) | | 70 (49-85) | | <0.001 |
| Physical Role Limitation | 0 (0-0) | 0 (0-25) | | 0 (0-75) | | 0.002 |
| Emotional Functioning | 48 (36-65) | 56 (42-69) | | 68 (52-80) | | 0.005 |
| Emotional role limitation | 33 (0-67) | 66 (0-75) | | 66 (33-100) | | 0.13 |
| Energy | 10 (5-27) | 30 (15-47) | | 35 (25-50) | | 0.0001 |
| Pain | 32 (32-45) | 57 (45-77) | | 67 (55-90) | | <0.001 |
| General Health | 45 (32-52) | 52 (31-66) | | 60 (49-75) | | 0.002 |
| Social functioning | 25 (12-44) | 50 (25-66) | | 62 (47-87) | | <0.001 |

Legend: MRC – Medical research council dyspnea score, SF36 - 36-Item Short Form Health Survey measuring health related quality of life in eight domains, lower scores represent worse health related functioning. Data are median (IQR) and compared using kruskall-wallis test.

**Supplemental table 3 – Univariate and multivariate models demonstration association of factors with self-reported chest pain**

|  | Unadjusted OR (95% CI) | P value | Adjusted OR (95% CI) | P value |
| --- | --- | --- | --- | --- |
| Age (years) | 0.97 (0.96-0.99) | <0.001 | 0.98 (0.96-0.99) | 0.02 |
| Male sex | 0.7 (0.43-1.12) | 0.14 | 0.82 (0.47-1.41) | 0.48 |
| Non Caucasian ethnicity | 1.36 (0.77-2.35) | 0.3 | 1.31 (0.67-2.55) | 0.42 |
| Time from symptom onset (weeks) | 1 (0.98-1.01) | 0.4 | 0.99 (0.97-1.005) | 0.23 |
| VOC period | 0.51 (0.33-0.8) | 0.003 | 0.4 (0.21-0.75) | 0.005 |
| Disease severity  Moderate  Severe | 0.43 (0.19-0.9)  0.51 (0.22-1.07) | 0.033  0.09 | 0.64 (0.26-1.49)  0.59 (0.19-1.63) | 0.32  0.33 |
| Vaccinated at time of infection | 0.95 (0.29-2.72) | 0.9 | 1.44 (0.39-4.74) | 0.55 |
| Vaccinated at time of review | 0.73 (0.47-1.13) | 0.2 | 1.05 (0.57-1.96) | 0.87 |

**Supplemental table 4 - Univariate and multivariate models demonstration association of factors with self-reported palpitations**

|  | Unadjusted OR (95% CI) | P value | Adjusted OR (95% CI) | P value |
| --- | --- | --- | --- | --- |
| Age (years) | 0.97 (0.96-0.99) | 0.02 | 0.99 (0.97-1.01) | 0.24 |
| Male sex | 0.72 (0.42-1.2) | 0.2 | 0.93 (0.49-1.7) | 0.82 |
| Non Caucasian ethnicity | 0.94 (0.49-1.73) | 0.9 | 1.06 (0.48-2.25) | 0.86 |
| Time from symptom onset (weeks) | 0.29 (0.16-0.5) | <0.001 | 0.99 (0.97-1.005) | 0.18 |
| VOC period | 0.99 (0.98-1.01) | 0.4 | 0.32 (0.14-0.65) | 0.002 |
| Disease severity  Moderate  Severe | 0.33 (0.11-0.78)  0.21 (0.05-0.61) | 0.022  0.011 | 0.41 (0.12-1.11)  0.32 (0.06-1.11) | 0.1  0.1 |
| Vaccinated at time of infection | 0.21 (0.01-1.04) | 0.13 | 0.45 (0.02-2.69) | 0.46 |
| Vaccinated at time of review | 0.46 (0.28-0.76) | 0.003 | 0.76 (0.38-1.51) | 0.44 |
